# Post-mortem magnetic resonance imaging in patients with suspected prion disease: Pathological confirmation, sensitivity, specificity and observer reliability. A national registry

**DOI:** 10.1371/journal.pone.0201434

**Published:** 2018-08-07

**Authors:** Lorna M. Gibson, Francesca M. Chappell, David Summers, Donald A. Collie, Robin Sellar, Jonathan Best, Richard Knight, James W. Ironside, Joanna M. Wardlaw

**Affiliations:** 1 Department of Clinical Radiology, New Royal Infirmary of Edinburgh, Edinburgh, United Kingdom; 2 Usher Institute of Population Health Sciences and Informatics, University of Edinburgh, Edinburgh, United Kingdom; 3 Centre for Clinical Brain Sciences and UK Dementia Research Institute at the University of Edinburgh, Edinburgh, United Kingdom; 4 Department of Neuroradiology, Western General Hospital, Edinburgh, United Kingdom; 5 Fife Acute Hospitals NHS Trust, Kirkcaldy, United Kingdom; 6 National Creutzfeldt-Jakob Disease Research and Surveillance Unit, Western General Hospital, Edinburgh, United Kingdom; 7 Brain Research Imaging Centre, Western General Hospital, Edinburgh, United Kingdom; FDA, UNITED STATES

## Abstract

The relationship between magnetic resonance imaging (MRI) and clinical variables in patients suspected to have Creutzfeldt-Jakob Disease (CJD) is uncertain. We aimed to determine which MRI features of CJD (positive or negative), previously described in vivo, accurately identify CJD, are most reliably detected, vary with disease duration, and whether combined clinical and imaging features increase diagnostic accuracy for CJD. Prospective patients suspected of having CJD were referred to the National CJD Research and Surveillance Unit between 1994–2004; post-mortem, brains were sent for MRI and histopathology. Two neuroradiologists independently assessed MRI for atrophy, white matter hyperintensities, and caudate, lentiform and pulvinar signals, blind to histopathological diagnosis and clinical details. We examined differences in variable frequencies using Fisher’s exact tests, and associations between variables and CJD in logistic regression models. Amongst 200 cases, 118 (59%) with a histopathological diagnosis of CJD and 82 (41%) with histopathological diagnoses other than CJD, a logistic regression model including age, disease duration at death, atrophy, white matter hyperintensities, bright or possibly bright caudate, and present pulvinar sign correctly classified 81% of cases as CJD versus not CJD. Pulvinar sign alone was not independently associated with an increased likelihood of histopathologically-confirmed CJD (of any subtype) or sporadic CJD after adjustment for age at death, disease duration, atrophy, white matter hyperintensities or caudate signal; despite the large sample, data sparsity precluded investigation of the association of pulvinar sign with variant CJD. No imaging feature varied significantly with disease duration. Of the positive CJD signs, neuroradiologists most frequently agreed on the presence or absence of atrophy (agreements in 169/200 cases [84.5%]). Combining patient age, and disease duration, with absence of atrophy and white matter hyperintensities and presence of increased caudate signal and pulvinar sign predicts CJD with good accuracy. Autopsy remains essential.

## Introduction

Creutzfeldt-Jakob disease (CJD) is a fatal neurological disease characterised by a post-translational conformational change in the prion protein. It most often occurs sporadically (sCJD), can be genetic, but importantly sometimes is iatrogenic (iCJD) or acquired by zoonotic infection (variant CJD [vCJD]) arising from dietary contamination with bovine spongiform encephalopathy[1]. Clinical diagnosis is based on the presence of key clinical features, the exclusion of other illnesses and supportive test findings, including magnetic resonance imaging (MRI)[2] and cerebrospinal fluid (CSF) protein analyses.

Various brain MRI features have been described in patients with CJD. Most notably these include increased signal in the caudate and lentiform nucleus in sCJD and, in vCJD, increased signal in the posterior third of the thalamus compared to other basal ganglia (named the pulvinar sign)[2]. However, CJD is rare. Annual mortality rates from sCJD range between 0.76 and 1.32 per million per year in the United Kingdom[3]. Variant CJD is even rarer, with only 231 cases reported worldwide between October 1996 and July 2017[4]. Most CJD presents in middle to late life when other neurodegenerative disorders, which may mimic CJD, are common, adding to the diagnostic difficulty. As most of the data concerning MRI findings in CJD are derived from routine clinical practice[2, 5], the relationship between the MRI findings and disease duration or other clinical features is uncertain. Some variation in reported imaging findings may reflect the point in the disease course at which the patient was imaged, as it is often difficult to undertake MRI in patients with advanced forms of neurodegenerative diseases.

The UK National CJD Research and Surveillance Unit (NCJDRSU) was established in May 1990. From that time onwards, brains of most patients suspected of having died of CJD in the UK were referred to the Unit for neuropathological analysis, with relatives giving consent for research, including MRI, in some cases. We aimed to determine, in patients with suspected CJD in life, whether there were specific (positive or negative) features, or combinations thereof on visual assessment that identified CJD from non-CJD, vCJD and sCJD at the point of death, if duration of disease influenced imaging appearances, and which imaging features were most reliably detected. We were able to achieve these aims using the unique post-mortem MRI dataset held by the NCJDRSU.

## Materials and methods

### Patients

The methodology of the UK CJD surveillance system is described elsewhere (*http://www.cjd.ed.ac.uk/sites/default/files/NCJDRSU%20surveillance%20protocol-april%202017%20rev2.pdf*). In brief, wherever possible, alive suspected cases referred to the NCJDRSU are clinically examined by the Unit neurologist who also reviews case records, records a detailed history, and takes relevant blood and CSF specimens. To determine a definite diagnosis in as many cases as possible, autopsy is encouraged and facilitated by transferring brain material to the NCJDRSU pathology laboratory. During the study period of 1994–2004, 1623 referrals were made to the NCJDRSU, for whom 184 whole brains and 178 half brains were available. Relatives gave written consent for research for 200 deceased suspected cases, resulting in our study sample of 112/184 (60.9%) of available whole brains, and 88/178 (49.4%) of available half brains. The ethics approval for the use of human tissues in the MRC Edinburgh Brain Bank for research was recently updated by the East of Scotland Research Ethics Service (reference: 16/ES/008). Separate local institutional approval for projects involving human tissue and related data is not required, but the relevant local institutions (i.e. the University of Edinburgh and NHS Lothian) are provided with details of the application and their support is required before the application is submitted to the relevant local ethics committee.

### Imaging

We fixed brain specimens in a 10% formalin solution for at least three weeks, then drained the formalin and dried the specimens before scanning. Post-mortem MRI signal characteristics are positively correlated with in-vivo measurements[6], and remain stable over long periods after fixation[7, 8]. We obtained T2 and proton density (PD) weighted MRI on one of three research-dedicated scanners, which were replaced over the 10-year study period. These included a 1.0T Siemens Magnetom from 21/10/1994 to 23/11/1998 (TR, TE, slice thickness: T2 2200, 90, 5 mm; PD 2200, 20, 5 mm), a 2.0T Elscint (now General Electric) Prestige from 08/12/1998 to 01/10/1999 (TR, TE, slice thickness: T2 fast spin echo (FSE) 4800, 128, 5 mm; PD FSE 4800, 16, 5 mm), and a 1.5T General Electric Signa Echospeed from 27/01/2000 onwards (TR, TE, slice thickness: T2 FSE 2000, 105, 3 mm; PD FSE 2000, 15, 3 mm). We implemented the MRI sequences to produce as similar brain signals as possible on each scanner, and each was optimized for visual assessment of T2 or PD images as in clinical practice. We tested different methods of scanning (in or out of fluid, different fluids, different sequences, scan duration scanning, and methods to minimise vibration artifact) to design the final protocol. Diffusion weighted images (DWI) were too degraded by large artefacts from air trapped within sulci or bubbles forming from air dissolved within fluid to be useful. We double wrapped brain specimens in plastic bags, and rested them on foam pads within the quadrature head coil on the inferior surface of the frontal lobe(s) and cerebellum/brain stem.

### Image reading

Two consultant neuroradiologists independently reviewed images at two separate time periods, blind to all clinical and pathological data and each other’s readings. Images were printed on to film for viewing on lightboxes. For each scanner, the images were printed on settings to give optimal T2 or PD tissue differentiation based on the standard T2 or PD parameters that were being used in ongoing in vivo studies and clinical exams that were being performed on the same scanners in the same time period, i.e. printed images were optimised for clinical radiological rating.

The neuroradiologists recorded the presence or absence of generalised cerebral atrophy and white matter hyperintensities (S1 File). They rated signals in the caudate nucleus, lentiform nucleus and posterior third of the thalamus (pulvinar) as bright, possibly bright or normal compared with expected normal signal in these structures based on experience and in relation to other brain tissues on the same scan. They rated pulvinar signal as brighter, the same, or less bright than that of the putamen, with brighter signal defining the ‘pulvinar sign.’ They rated all signals separately on T2 and PD images. After these ratings, they recorded their overall judgement of the diagnosis as either CJD (subtyped in to variant or sporadic), or not CJD.

### Neuropathological and clinical data

Neuropathological examinations were performed after brain specimens were imaged. We classified cases of CJD according to standardised criteria used across Europe (http://www.cjd.ed.ac.uk/sites/default/files/criteria_0.pdf) which detail that neuropathological confirmation is required for definitive diagnosis. The methods used for neuropathological diagnosis are summarized elsewhere (www.cjd.ed.ac.uk/sites/default/files/neuropath.pdf). All cases underwent the same histopathological examinations for CJD and non-CJD diagnoses including additional methods as necessary after initial examination. Thus in non-CJD tissue-based diagnosis was made using the relevant clinical standard histopathological methods.

We used available data on age at death and disease duration from clinical records held at the NCJDRSU. We collected these data prospectively from several sources: direct interview of relatives (and patient, if possible) at the time of referral to the NCJDRSU, medical records, and death certificates.

### Statistical analyses

We designated one reader’s (JMW) diagnoses as the reference point and compared these to neuropathological diagnoses to determine the cases for which correct diagnoses were reached. We present descriptive statistics of the cohorts’ clinical, neuropathological and imaging data. We used a two-tailed Fisher’s exact test to examine for significant differences in the frequencies of nominal imaging variables between two groups. We used an independent t-test to examine for significant differences in the mean of continuous variables between two groups.

We used logistic regression analyses to identify imaging features which may be associated with CJD compared to not CJD, vCJD compared to not vCJD, and sCJD compared to not sCJD. We considered the following variables: brain atrophy, white matter hyperintensities, and ratings of T2 signals of the caudate and lentiform nuclei, and presence of the pulvinar sign. We excluded variables with low frequencies, or if there were strong relationships between predictors, as regression assumes independence. There was a strong relationship between caudate signal on T2 and lentiform signal on T2, and as fewer data were available for lentiform ratings, this was excluded from all three models. No patients with vCJD were rated as having atrophy or white matter signal change, therefore we excluded these two variables from our model of vCJD compared to not vCJD. We assessed the calibration of each model using area under the curve.

To assess agreement between observers, and between observers’ final diagnoses and pathological diagnoses, we report percentage of cases agreed on, rather than Kappa which is dependent on prevalence. We used SPSS Statistics (version 22.0.0.1, New York, United States of America) for statistical analyses.

## Results

We imaged 200 brains between 1994 and 2004, of whom 118 (59%) had neuropathological diagnoses of CJD, 32 (16%) with vCJD, 75 (38%) with sCJD, seven (4%) with iatrogenic CJD and four (2%) with familial CJD. The remainder had non-CJD diagnoses (Table 1). Whole brains were available from 112/200 patients (56%), with half brains available from the remaining patients (Table 2).

**Table 1:** Included patients.

Diagnosis			n	Age at death Mean (SD)^a^	Disease duration in months Mean (SD)^a^
**CJD**			**118**	54 (20)	10 (9)
	**Sporadic**		75	67 (9)	7 (6)
	**Variant**		32	27 (8)	16 (8)
	**Iatrogenic**		7		
	**Familial**		4		
**Non-CJD**			**82**	62 (22)	3 (10)
	**Neurodegenerative**		**42**	64 (23)	3 (10)
		Alzheimer disease	28		
		Lewy body dementia	12		
		Alzheimer with Lewy body	2		
	**Other**		**15**	50 (21)	3 (8)
		Oedema	5		
		Encephalopathy	3		
		Age related changes	2		
		Chronic subcortical encephalitis	1		
		John Cunningham virus	1		
		Motor neurone disease	1		
		Prader-Willi syndrome	1		
		Viral encephalitis	1		
	**Vascular**		**11**	66 (25)	1 (5)
		Infarct	7		
		Ischaemia	2		
		Amyloid angiopathy	1		
		Arteriovenous malformation	1		
	**Neoplastic**		**6**	72 (6)	1 (2)
		Brain metastases	2		
		Ependymoma	1		
		Lymphoma	2		
		Paraneoplastic syndrome	1		
	**Multiple**		**5**	68 (6)	0 (0)
		Alzheimer and amyloid angiopathy	1		
		Alzheimer and Parkinson disease	1		
		Infarct and small cell carcinoma	1		
		Infarcts and meningoencephalitis	1		
		Lewy body and vascular malformation	1		
	**Demyelination**		**3**	58 (7)	19 (19)
		Multifocal demyelination	1		
		Multiple sclerosis	1		
		Non-specified demyelination	1		

SD = standard deviation, CJD = Creutzfeldt-Jakob Disease

^**a**^ Mean and standard deviation presented for summary groups only

**Table 2:** Reference reader signal ratings vs pathology.

	CJD (all types)^a^ n (%)	Not CJD^b^ n (%)	p value	vCJD n (%)	sCJD n (%)	p value
n	118	82		32	75	
Whole brain	68 (57.6)	44 (53.7)		11 (34.4)	47 (62.7)	
Correctly identified by neuroradiologist	89 (75.4)	56 (68.3)		20 (62.5)	28 (37.3)	
Brain atrophy	13 (11.0)	31 (37.8)	<0.0001	0 (0.0)	11 (14.7)	0.032
White matter hyperintensities	16 (13.6)	39 (47.6)	<0.0001	0 (0.0)	15 (20.0)	0.005
**Caudate nucleus signal**						
Bright on T2	60 (50.9)	11 (13.4)	<0.0001	16 (50.0)	39 (52.0)	1.000
Bright or possibly bright on T2	85 (72.0)	20 (24.4)	<0.0001	24 (75.0)	54 (72.0)	0.816
Bright on PD	107 (90.7)	73 (89.0)	0.812	31 (96.9)	68 (90.7)	0.431
Bright or possibly bright on PD	111 (94.1)	76 (92.7)	0.774	32 (100.0)	70 (93.3)	0.319
**Lentiform nucleus signal**						
Bright on T2	69 (58.5)	14 (17.1)	<0.0001	20 (62.5)	43 (57.3)	0.672
Bright or possibly bright on T2	95 (80.5)	28 (34.2)	<0.0001	29 (90.6)	58 (77.3)	0.174
Bright on PD	105 (89.0)	69 (84.2)	0.393	27 (84.4)	69 (92.0)	0.299
Bright or possibly bright on PD	112 (94.9)	75 (91.5)	0.388	31 (96.9)	71 (94.7)	1.000
**Pulvinar signal**						
Bright on T2	38 (32.2)	6 (7.3)	<0.0001	15 (46.9)	19 (25.3)	0.041
Bright or possibly bright on T2	53 (44.9)	9 (11.0)	<0.0001	20 (62.5)	28 (37.3)	0.020
Bright on PD	89 (75.4)	50 (61.0)	0.042	30 (93.8)	54 (72.0)	0.011
Bright or possibly bright on PD	101 (85.6)	57 (69.5)	0.008	31 (96.9)	61 (81.3)	0.036
**Pulvinar sign**						
Present on T2	8 (9.8)	3 (2.5)	0.530	6 (18.8)	2 (2.7)	0.009
Present or pulvinar signal same as putamen on T2	49 (41.5)	17 (20.7)	0.002	20 (62.5)	25 (33.3)	0.010
Present on PD	10 (8.5)	3 (3.7)	0.246	8 (25.0)	2 (2.7)	0.001
Present or pulvinar signal same as putamen on PD	91 (77.1)	49 (59.8)	0.012	29 (90.6)	55 (73.3)	0.070

CJD = Creutzfeldt-Jakob Disease, vCJD = new variant Creutzfeldt-Jakob Disease, sCJD = sporadic Creutzfeldt-Jakob Disease, PD = proton density

Tests: Fisher’s exact 2 sided

^**a**^ There were four cases of familial and seven cases of iatrogenic CJD included in the cohort

^**b**^ Six non-CJD cases do not have age at death data

Patients with CJD were significantly younger at death than patients without CJD (mean age 54 (standard deviation [SD] 20) vs 62 [SD 22] years, p = 0.004) and had significantly longer disease duration (mean 10 [SD 9] vs 3 [SD 10] months, p<0.0001). Patients with vCJD were significantly younger than those with sCJD at death (mean age 27 [SD 8] vs 67 [SD 9] years, p<0.0001) and had significantly longer disease duration (mean 16 [SD 8] vs 7 [SD 6] months, p<0.0001).

### Signal changes: CJD vs not CJD

**Fig 1:**
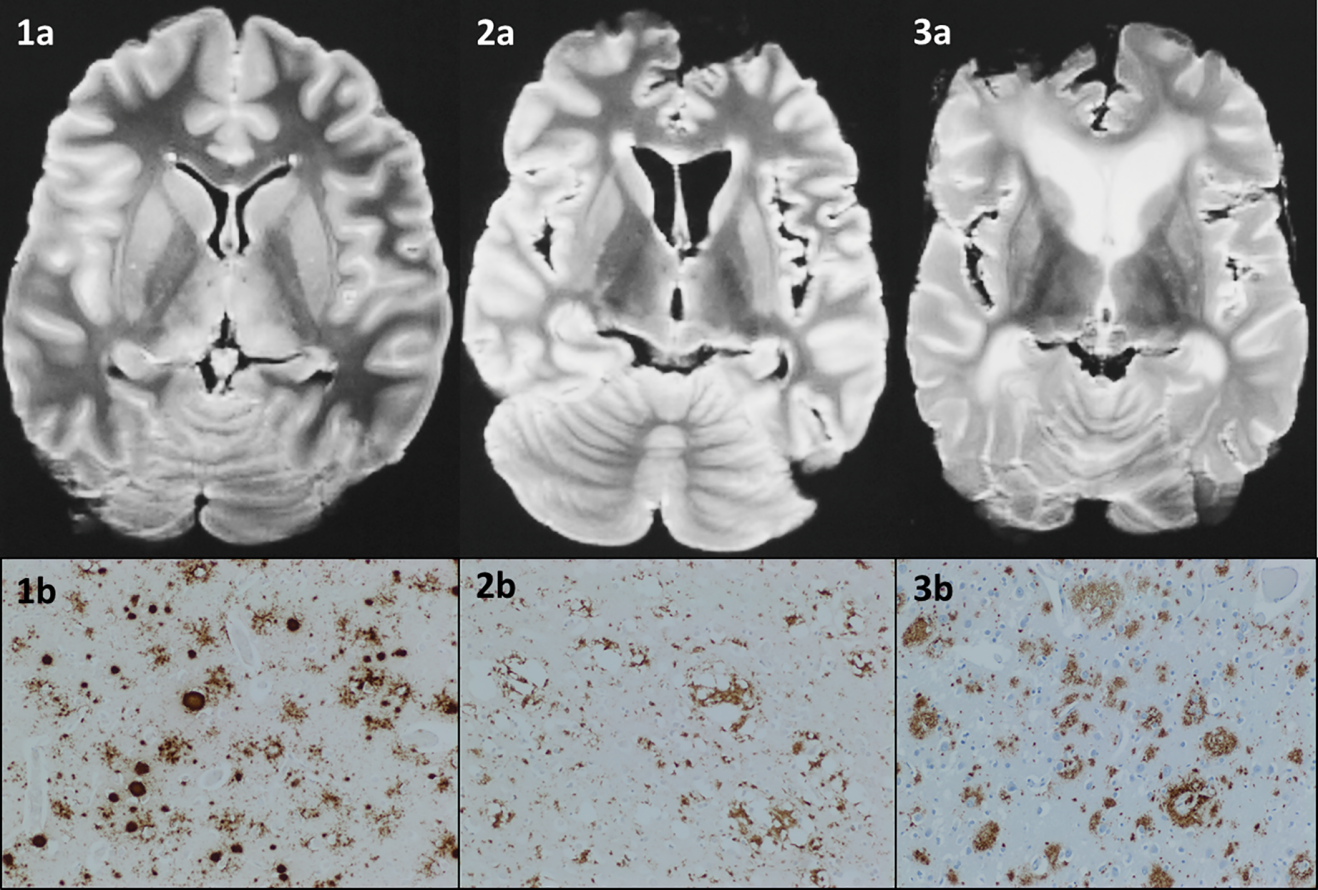
Examples of patients with vCJD, sCJD and non-CJD diagnoses. Axial T2-weighted images of post-mortem brains from patients with suspected CJD, imaged using 1.0T Siemens Magnetom, with corresponding immunohistochemistry. 1a) Correctly diagnosed by the reference reader as variant CJD, with all basal ganglia rated as bright, without atrophy or white matter hyperintensities. 1b) Immunohistochemistry for prion protein in the frontal cortex in variant CJD shows dense staining (brown) of rounded florid plaques, with additional microplaques and pericellular deposits also demonstrated (12F10 antibody, x 100). 2a) Correctly diagnosed by the reference reader as sCJD, with only the caudate nuclei rated as bright, without a bright pulvinar, or atrophy or white matter hyperintensities. 2b) Immunohistochemistry for prion protein in the frontal cortex in sporadic CJD (MM2 subtype) shows dense deposition (brown) around areas of confluent spongiform change (12F10 antibody, x 100). 3a) Correctly diagnosed by the reference reader as non-CJD without any basal ganglia signal change, but with atrophy and white matter hyperintensities present. 3b) Immunohistochemistry for Aβ in the frontal cortex in Alzheimer’s disease shows numerous plaques (brown), including a cored plaque (lower right) (6F/3D antibody, x 100).

Compared to patients without CJD, patients with CJD were less frequently rated as having cerebral atrophy (13/118, 11% vs 31/82, 38%, p<0.0001), and white matter hyperintensities (16/118, 14% vs 39/82, 48%, p<0.0001). Compared to patients without CJD, patients with CJD were significantly more frequently rated to have bright or possibly bright caudate and lentiform nuclei, and present pulvinar sign on T2 (Fig 1 and Table 2).

A binomial logistic regression model which included patient and imaging variables correctly classified 81% of cases as CJD or not CJD. The odds of CJD were significantly increased with increased disease duration (odds ratio (OR) 1.129, 95% confidence interval (CI) 1.071–1.191, p<0.0001), and bright or possibly bright caudate on T2 (OR 7.854, 95% CI 3.656–16.874, p<0.0001) and significantly reduced by the presence of atrophy (OR 0.255, 95% CI 0.091–0.713, p = 0.009) (Table 3).

**Table 3:** Logistic regression models.

	CJD compared to not CJD^a^				vCJD compared to not vCJD^b^				sCJD compared to not sCJD^c^			
		95% confidence interval				95% confidence interval				95% confidence interval		
Variable	Odds ratio	Lower	Upper	p value	Odds ratio	Lower	Upper	p value	Odds ratio	Lower	Upper	p value
Age at death	1.102	0.905	1.340	0.334	0.420	0.310	0.570	<0.0001	2.662	1.954	3.625	<0.0001
Disease duration	1.129	1.071	1.191	<0.0001	1.103	1.049	1.161	<0.0001	1.071	1.024	1.119	0.002
Brain atrophy^d^	0.255	0.091	0.713	0.009	-	-	-	-	0.360	0.130	0.992	0.048
White matter signal change^**d**^	0.260	0.103	0.658	0.004	-	-	-	-	0.326	0.124	0.858	0.024
Bright or possibly bright caudate on T2	7.854	3.656	16.874	<0.0001	3.325	0.958	11.536	0.058	7.421	3.292	16.731	<0.0001
Pulvinar sign^d^	3.306	0.674	16.207	0.140	-	-	-	-	0.729	0.115	4.605	0.737

CJD = Creutzfeldt-Jakob disease, vCJD = variant CJD, sCJD = sporadic CJD

^**a**^ Area under the curve = 0.881 (95% confidence interval 0.831–0.931), p-value <0.0001.

^**b**^ Area under the curve = 0.950 (95% confidence interval 0.921–0.978), p-value <0.0001.

^**c**^ Area under the curve = 0.860 (95% confidence interval 0.809–0.911), p-value <0.0001.

^**d**^ These variables were excluded from the model of vCJD compared to not vCJD due to lack of data (see also S1 Table)

### Signal changes: With subtypes of CJD

Compared to patients with sCJD, patients with vCJD were rated significantly more frequently as having bright or possibly bright pulvinar signals on T2 and PD, and present pulvinar sign on T2 (Table 2).

Brain atrophy, white matter hyperintensity and pulvinar sign variables were excluded from a binomial logistic regression model to predict vCJD compared to not vCJD due to data sparsity (Table 3, S1 Table). The model included age at death, disease duration and bright or possibly bright caudate on T2, and correctly classified 90% of cases as vCJD or not vCJD. Increased age at death significantly reduced the odds of vCJD (OR 0.420, 95% CI 0.310–0.570, p<0.0001), whereas increased disease duration significantly increased the odds of vCJD (OR 1.103, 95% CI 1.049–1.161, p<0.0001) (Table 3).

A final binomial logistic regression model correctly classified 82% of cases as sCJD or not sCJD. The odds of sCJD increased significantly with increased age (OR 2.662, 95% CI 1.954–3.625, p<0.0001) and disease duration (OR 1.071, 95% CI 1.024–1.119, p = 0.002), and presence of bright or possibly bright caudate signal on T2 (OR 7.421, 95% CI 3.292–16.731, p<0.0001). Presence of atrophy and white matter hyperintensities significantly reduced the odds of sCJD (OR 0.360, 95% CI 0.130–0.992, p = 0.048; OR 0.326, 95% CI 0.124–0.858, p = 0.024 respectively) (Table 3).

Presence of the pulvinar sign did not significantly increase the odds of CJD or sCJD independently of the other factors included in the models (Table 3).

### Signal changes in CJD by duration of disease

The mean duration of disease amongst the 118 patients with CJD was 10 months. There was no significant difference in the frequency of any imaging variable in patients with disease duration of 10 months or less, compared to those with disease duration greater than 11 months.

### Diagnostic accuracy of signs

No individual sign had over 80% sensitivity and specificity for detecting CJD versus not CJD, or for differentiating vCJD from sCJD (S2 Table). For predicting either vCJD or sCJD, bright or possibly bright caudate on PD, and bright or possibly bright pulvinar on PD had sensitivities of over 80%. Bright pulvinar sign on T2 had 97.3% and 81.3% specificity for vCJD and sCJD respectively, but less than 40% sensitivity for either (S2 Table).

### Observer agreement

Observers most frequently agreed on the presence or absence of brain atrophy (agreements in 169/200 cases (84.5%)). Observers least frequently agreed on the rating of caudate signal on PD as bright compared to possibly bright or not bright (agreed on 50/200 cases (25.0%)). Observers agreed on final diagnoses in 102/200 (51.0%) of cases (S3 Table).

Comparing one reader’s (JMW) overall judgement of final diagnosis to the histopathological diagnosis resulted in agreement on the presence or absence of CJD in 145/200 (72.5%) cases (Table 4).

**Table 4:** Misdiagnoses.

	Cases of CJD			Cases of non-CJD		
	Correctly identified CJD (n = 89) n (%)	Missed CJD (n = 29) n (%)	p value	Correctly identified not CJD (n = 56) n (%)	Overcalled CJD (n = 26) n (%)	p value
Whole brains	54 (60.7)	14 (48.3)	0.282	31 (55.6)	13 (50.0)	0.812
Scanner 1 (1.0 tesla)	49 (55.1)	18 (62.1)	0.258	36 (64.3)	11 (42.3)	0.032
Scanner 2 (2.0 tesla)	12 (13.5)	6 (20.7)		8 (14.3)	2 (7.7)	
Scanner 3 (1.5 tesla)	28 (31.5)	5 (17.2)		12 (21.4)	13 (50.0)	
Mean age at death in years (SD)	52 (20)	57 (21)	0.246	65 (20)	55 (25)	0.053
Mean disease duration in months (SD)	9 (7)	13 (12)	0.012	3 (11)	3 (7)	0.699
Brain atrophy	6 (6.7)	7 (24.1)	0.016	21 (37.5)	10 (38.5)	1.000
White matter hyperintensities	9 (10.1)	7 (24.1)	0.067	31 (55.4)	8 (30.8)	0.057
**Caudate nucleus signal**						
Bright on T2	57 (64.1)	3 (10.4)	<0.0001	0 (0.0)	11 (42.3)	<0.0001
Bright or possibly bright on T2	76 (85.4)	9 (31.0)	<0.0001	5 (8.9)	15 (57.7)	<0.0001
Bright on PD	87 (97.8)	20 (69.0)	<0.0001	47 (83.9)	26 (100.0)	0.051
Bright or possibly bright on PD	88 (98.9)	23 (79.3)	0.001	50 (89.3)	26 (100.0)	0.170
**Lentiform nucleus signal**						
Bright on T2	65 (73.0)	4 (13.8)	<0.0001	1 (1.8)	13 (50.0)	<0.0001
Bright or possibly bright on T2	85 (95.5)	10 (34.5)	<0.0001	10 (17.9)	18 (69.2)	<0.0001
Bright on PD	83 (93.3)	22 (75.9)	0.016	45 (80.4)	24 (92.3)	0.209
Bright or possibly bright on PD	88 (98.9)	24 (82.8)	0.003	50 (89.3)	25 (96.2)	0.422
**Pulvinar signal**						
Bright on T2	37 (41.6)	1 (3.5)	<0.0001	1 (1.8)	5 (19.2)	0.011
Bright or possibly bright on T2	49 (55.1)	4 (13.8)	<0.0001	1 (1.8)	8 (30.8)	<0.0001
Bright on PD	75 (84.3)	14 (48.3)	<0.0001	27 (48.2)	23 (88.5)	0.001
Bright or possibly bright on PD	79 (88.8)	22 (75.9)	0.125	34 (60.7)	23 (88.5)	0.011
**Pulvinar sign**						
Present on T2	6 (6.7)	2 (7.0)	1.000	2 (3.6)	1 (3.9)	1.000
Present or same signal as putamen on T2	42 (47.2)	7 (24.1)	0.032	9 (16.1)	8 (30.8)	0.150
Present on PD	8 (9.0)	2 (6.9)	1.000	2 (3.6)	1 (3.9)	1.000
Present or same signal as putamen on PD	72 (80.9)	19 (65.5)	0.125	27 (48.2)	22 (84.6)	0.002

CJD = Creutzfeldt-Jakob disease, SD = standard deviation, PD = proton density

Comparing two readers’ (JMW and RS) overall judgements of final diagnosis resulted in agreement of presence or absence of CJD in 102/200 (51%) cases (S3 Table).

### Misdiagnosed cases

There were no significant differences in the availability of whole brains or mean age at death in patients who were correctly diagnosed with CJD to those who had a diagnosis of CJD missed by the reference reader, or in patients in whom CJD was overcalled by the reference reader compared to those in whom a non-CJD diagnosis was correctly made (Table 4). Patients who were correctly diagnosed with CJD had a significantly shorter duration of disease compared to those in whom a diagnosis of CJD was missed (9 [SD 7] months vs 13 [SD 1]) months, p = 0.012). There was a significant difference regarding which scanner was used for imaging in patients in whom CJD was overcalled by the reference reader compared to those in whom a non-CJD diagnosis was correctly made (Table 4).

#### Missed CJD

The reference reader missed 29 cases of CJD (four vCJD, 21 sCJD, and two cases each of familial and iatrogenic CJD) (Table 4). Compared to patients in whom a diagnosis of CJD was correctly made, missed cases were significantly more frequently rated as having brain atrophy, and significantly less frequently rated as having bright caudate nuclei on T2 and PD, bright lentiform nuclei on T2 and PD, and bright pulvinar on T2 and PD (Table 4). There was no difference in the proportion rated as having bright pulvinar sign on either T2 or PD between these two groups (Table 4).

#### Overcalled CJD

The reference reader incorrectly diagnosed CJD in 26 patients. Compared to patients in whom a non-CJD diagnosis was correctly made, overcalled cases were significantly more frequently rated as having bright caudate and lentiform nuclei on T2, bright pulvinar nuclei on T2 and PD, and bright pulvinar sign on PD (Table 4).

## Discussion

We found that brain atrophy and white matter hyperintensities are less frequently present in patients with CJD, and the presence of bright caudate increases the likelihood of a final diagnosis of CJD, in this large dataset. Models including patient variables and imaging signs predicted most CJD versus no CJD, sCJD versus not sCJD, and vCJD versus not vCJD very accurately. Interobserver agreement regarding MRI signal changes varied, and final diagnoses based on radiologists’ overall judgments of the imaging agreed with pathological diagnoses in 145/200 (72.5%) cases. If this experiment were repeated by radiologists who were informed of our imaging feature results and provided with information on disease duration and age (as would be available in clinical practice), that diagnostic accuracy would likely improve further. Missed cases of CJD were less likely to have bright basal ganglia, indicating that even at death (i.e. end-stage disease), signs thought characteristic of CJD and useful in pre-mortem investigations are not 100% sensitive or specific for CJD post-mortem, and, as our logistic regression models suggest, combinations of patient variables and presence or absence of imaging variables identify most CJD cases, suggesting that, in contrast to some studies, positive individual imaging signs should not be used alone[5, 9, 10], and useful negative signs also should be sought to differentiate CJD from non-CJD. Whilst autopsy rates remain extremely low, neuropathology remains essential for a definitive diagnosis of CJD.

To our knowledge, this study represents one of the largest post-mortem MRI dataset with pathological correlation in any disease, the largest post-mortem imaging study of patients with suspected CJD, and is comparable in size to the largest pre-mortem imaging studies of patients with suspected CJD[9–11]. Three large national surveillance programmes have published data on cohorts of patients with suspected CJD[10, 12, 13] but none routinely performed or have published data on post-mortem imaging.

This study also benefits from consistent data collection on a rare disease due to the nationwide referral system to the NCJDRSU. The study therefore comprises the largest collection of neuropathological and neuroimaged brains from unselected patients with suspected CJD, referred by doctors working across an entire country of approximately 60 million people. The cohort is entirely representative of, and therefore data are directly relevant to, patients encountered in routine clinical practice. Compared to in vivo studies of imaging findings performed in selected groups of CJD patients[5, 9] results from our study are more generalisable to routine clinical practice where firm diagnosis is yet to be established in patients either late in the disease process or during virtual autopsy.

Our research MRI scanner hardware was replaced three times during the 10-year study. Whilst using different scanners undoubtedly creates noise in the dataset, this likely has a limited effect on the imaging outcome measures, which were derived by visual assessment of standard structural images rather than quantitative measures, as found by others[10]. As hundreds of different scanners would be used nationally or internationally to image patients with suspected CJD, our results are generalisable to routine practice. Our T2-weighted imaging produces similar images to fluid-attenuated inversion recovery (FLAIR) sequences which are commonly used in pre-mortem imaging of CJD due to the low signal intraventricular air in our post-mortem specimens. Whilst visual assessment of images may seem simple compared to digital image analysis techniques, most diagnoses of CJD on imaging in clinical practice are based on visual assessment, and our imaging outcomes based on visual assessment are immediately applicable to both clinical practice and to research studies performed using different MRI scanners.

The radiologists were blinded to clinical and pathological data, reducing the impact of these on imaging ratings and opinions on final diagnoses. The majority of samples were whole brains. Availability of only hemi-brains may have reduced accuracy in these 88/200 (44.0%) patients, particularly if parasagittal nuclei close to tissue-fluid interfaces were affected by artefact, or if lack of a second side affected observers’ certainty regarding signal characteristics. However, there was no evidence of altered accuracy within our sample, as there was no significant association between the availability of whole rather than hemi-brains and the correct identification of either CJD (p = 0.282) or non-CJD (p = 0.812) (Table 4). The effect of fixation on signal characteristics is likely to be minimal, particularly T2 signal characteristics of basal ganglia, which change slowly, even over extended periods[7], and is even less relevant for measures of relative, rather than absolute, signal changes as used in this study.

Interobserver agreement levels between the two readers, both of whom are experts in the field, were entirely consistent with other studies of expert neuroradiologists’ agreement on final diagnoses of CJD and presence/absence of imaging features based on visual inspection of images[10, 14]. Whilst it appeared that the most recent scanner was associated with increased overcalling CJD, this is likely a confounder, as overcalling rates did not increase with increased magnet field strength.

Hospital autopsy rates in the UK have fallen significantly[15] prompting a search for non- or minimally invasive examination strategies[16], and forensic radiology is a rapidly growing subspecialty[17]. However, major discrepancies in causes of death identified by consensus radiology reads of post-mortem MRI compared to autopsy occur in 43% of cases (95% CI 36–50%)[17], and post-mortem neuroimaging in 57 unselected cases demonstrated sensitivities ranging from 0 to 100% for the detection of relevant post-mortem findings[18].

This study of the largest cohort of post-mortem imaging in suspected CJD demonstrates that whilst using combinations of patient and imaging variables can accurately classify non-CJD and CJD subtypes, no individual or combination of imaging signs is 100% sensitive or specific, and autopsy remains essential for a definitive diagnosis of human prion disease.

## Supporting information

S1 Table Variables considered for inclusion in logistic regression models. CJD = Creutzfeldt-Jakob Disease, sCJD = sporadic CJD, vCJD = variant CJD, SD = standard deviation. Numbers represent frequencies unless otherwise specified.
(DOCX)

S2 Table Sensitivity, specificity, positive and negative predictive values of imaging characteristics for predicting CJD, vCJD and sCJD. sCJD = sporadic Creutzfeldt-Jakob disease, vCJD = variant Creutzfeldt-Jakob disease, CI = confidence interval, PD = proton density,—values cannot be calculated.
(DOCX)

S3 Table Interobserver agreement. PD = proton density, CJD = Creutzfeldt-Jakob Disease ^**a**^ The second reader did not provide a rating for pulvinar signal on T2 or PD for one case each; denominator is 199.
(DOCX)

S1 File Imaging coding for imaging readers. (DOCX)

S2 File Dataset. (SAV)
