# Supplementary material for: Post-mortem magnetic resonance imaging in patients with suspected prion disease: Pathological confirmation, sensitivity, specificity and observer reliability. A national registry
Source: PLoS One. 2018 Aug 7;13(8):e0201434. doi: 10.1371/journal.pone.0201434 (PMC6080765; doi:10.1371/journal.pone.0201434)
Supplement: S1 Table — CJD = Creutzfeldt-Jakob Disease, sCJD = sporadic CJD, vCJD = variant CJD, SD = standard deviation. Numbers represent frequencies unless otherwise specified. (DOCX) [file pone.0201434.s001.docx]

**S1 Table: Variables considered for inclusion in logistic regression models**

|  | **CJD compared to not CJD** | | **sCJD compared to not sCJD** | | **vCJD compared to not vCJD** | |
| --- | --- | --- | --- | --- | --- | --- |
|  | **Not CJD** | **CJD** | **Not sCJD** | **sCJD** | **Not vCJD** | **vCJD** |
|  |  |  |  |  |  |  |
| **Mean age in decades (range, SD)** | 6.2  (0 - 9.2, 2.2) | 5.4  (1.4 – 8.6, 2.0) | 5.1  (0 – 9.2, 2.5) | 6.7  (3.9 – 8.6, 0.9) | 6.3  (0 – 9.2, 1.8) | 2.7  (1.4 -5.1, 0.8) |
| **Mean disease duration in months (range, SD)** | 3.1  (0 - 53, 9.6) | 9.6  (0 – 39, 8.7) | 7.2  (0 – 53, 11.1) | 6.6  (1 – 35, 6.5) | 5.2  (0 – 53, 8.9) | 16.2  (7 – 39, 8.1) |
|  |  |  |  |  |  |  |
| **Brain atrophy** |  |  |  |  |  |  |
| Absent | 51 | 105 | 92 | 64 | 124 | 32 |
| Present | 31 | 13 | 33 | 11 | 44 | 0 |
|  |  |  |  |  |  |  |
| **White matter hyperintensities** |  |  |  |  |  |  |
| Absent | 43 | 102 | 85 | 60 | 113 | 32 |
| Present | 39 | 16 | 40 | 15 | 55 | 0 |
|  |  |  |  |  |  |  |
| **Caudate nucleus signal on T2** |  |  |  |  |  |  |
| Normal | 62 | 33 | 74 | 21 | 87 | 8 |
| Bright or possibly bright | 20 | 85 | 51 | 54 | 81 | 24 |
|  |  |  |  |  |  |  |
| **Lentiform nucleus signal on T2** |  |  |  |  |  |  |
| Normal | 54 | 23 | 60 | 17 | 74 | 3 |
| Bright or possibly bright | 28 | 95 | 65 | 58 | 94 | 29 |
|  |  |  |  |  |  |  |
| **Pulvinar sign** |  |  |  |  |  |  |
| Absent | 79 | 110 | 116 | 73 | 163 | 26 |
| Present | 3 | 8 | 9 | 2 | 5 | 6 |

CJD = Creutzfeldt-Jakob Disease, sCJD = sporadic CJD, vCJD = variant CJD, SD = standard deviation

Numbers represent frequencies unless otherwise specified
