# Supplementary material for: Post-mortem magnetic resonance imaging in patients with suspected prion disease: Pathological confirmation, sensitivity, specificity and observer reliability. A national registry
Source: PLoS One. 2018 Aug 7;13(8):e0201434. doi: 10.1371/journal.pone.0201434 (PMC6080765; doi:10.1371/journal.pone.0201434)
Supplement: S2 Table — sCJD = sporadic Creutzfeldt-Jakob disease, vCJD = variant Creutzfeldt-Jakob disease, CI = confidence interval, PD = proton density,—values cannot be calculated. (DOCX) [file pone.0201434.s002.docx]

**S2 Table: Sensitivity, specificity, positive and negative predictive values of imaging characteristics for predicting CJD, vCJD and sCJD.**

| **Predicting CJD** | **Sensitivity %**  **(95% CI)** | **Specificity %**  **(95% CI)** | **Positive predictive value**  **(95% CI)** | **Negative predictive value**  **(95% CI)** |
| --- | --- | --- | --- | --- |
| Brain atrophy | 11.0 (6.0 – 18.1) | 62.2 (50.8 – 72.7) | 29.6 (16.7 – 45.2) | 32.7 (25.4 – 40.7) |
| White matter hyperintensities | 13.6 (8.0 – 21.1) | 52.4 (41.1 – 63.6) | 29.1 (17.6 – 42.9) | 29.7 (22.4 – 37.8) |
| **Caudate nucleus signal** |  |  |  |  |
| Bright on T2 | 50.9 (41.9 – 60.2) | 86.6 (77.3 – 93.1) | 84.5 (74.0 – 92.0) | 55.0 (46.0 – 63.8) |
| Bright or possibly bright on T2 | 72.0 (63.0 – 79.9) | 75.6 (64.9 – 84.4) | 81.0 (72.1 – 88.0) | 65.3 (54.8 – 74.7) |
| Bright on PD | 90.7 (83.9 – 95.3) | 11.0 (5.1 – 19.8) | 59.4 (51.9 – 66.7) | 45.0 (23.1 – 68.5) |
| Bright or possibly bright on PD | 94.1 (88.2 – 97.6) | 7.3 (2.7 – 15.3) | 59.4 (51.2 – 66.5) | 46.2 (19.2 – 74.9) |
| **Lentiform nucleus signal** |  |  |  |  |
| Bright on T2 | 58.5 (49.0 – 67.5) | 82.9 (73.0 – 90.3) | 83.1 (73.3 – 90.5) | 58.1 (48.6 – 67.2) |
| Bright or possibly bright on T2 | 80.5 (72.2 – 87.2) | 65.9 (54.6 – 76.0) | 77.2 (68.8 – 84.3) | 70.1 (58.6 – 80.0) |
| Bright on PD | 89.0 (81.9 – 94.0) | 15.9 (8.7 – 25.6) | 60.3 (52.7 – 67.7) | 50.0 (29.9 – 70.1) |
| Bright or possibly bright on PD | 94.9 (89.3 – 98.1) | 8.5 (3.5 – 16.8) | 59.9 (52.5 – 67.0) | 53.9 (25.1 – 80.8) |
| **Pulvinar signal** |  |  |  |  |
| Bright on T2 | 32.2 (23.9 – 41.1) | 92.7 (84.8 – 97.3) | 86.4 (72.7 – 94.8) | 48.7 (40.7 – 56.8) |
| Bright or possibly bright on T2 | 44.9 (35.8 – 54.3) | 89.0 (80.2 – 94.9) | 85.5 (74.2 – 93.1) | 52.9 (44.2 – 61.5) |
| Bright on PD | 75.4 (66.7 – 82.9) | 39.0 (28.4 – 50.4) | 64.0 (55.5 – 72.0) | 52.5 (39.3 – 65.4) |
| Bright or possibly bright on PD | 85.6 (77.9 – 91.4) | 30.5 (20.8 – 41.6) | 63.9 (55.9 – 71.4) | 59.5 (43.3 – 74.4) |
| **Pulvinar sign** |  |  |  |  |
| Present on T2 | 10.2 (5.4 – 17.1) | 96.3 (89.7 – 99.2) | 80.0 (51.9 – 95.7) | 42.7 (35.5 – 50.2) |
| Present or same signal as putamen on T2 | 47.5 (38.2 – 56.9) | 79.3 (68.9 – 87.4) | 76.7 (65.4 – 60.2) | 51.2 (42.2 – 60.2) |
| Present on PD | 68.6 (59.5 – 76.9) | 43.9 (33.0 – 55.3) | 63.8 (54.8 – 72.1) | 49.3 (37.4 – 61.3) |
| Present or same signal as putamen on PD | 77.1 (68.5 – 84.4) | 40.2 (29.6 – 51.7) | 65.0 (56.5 – 72.9) | 55.0 (41.6 – 67.9) |

sCJD = sporadic Creutzfeldt-Jakob disease, CI = confidence interval, PD = proton density, - values cannot be calculated

| **Predicting vCJD** | Sensitivity %(95% CI) | Specificity %(95% CI) | Positive predictive value(95% CI) | Negative predictive value(95% CI) |
| --- | --- | --- | --- | --- |
| Brain atrophy | 0.0 (0.0 – 10.9) | 85.3 (75.3 – 92.4) | 0.0 (0.0 – 28.5) | 66.7 (56.3 – 76.0) |
| White matter hyperintensities | 0.0 (0.0 – 10.9) | 80.0 (69.2 – 88.4) | 0.0 (0.0 – 21.8) | 65.2 (54.6 – 74.9) |
| **Caudate nucleus signal** |  |  |  |  |
| Bright on T2 | 50.0 (31.9 – 68.1) | 48.0 (36.3 – 59.9) | 29.1 (17.6 – 42.9) | 69.2 (54.9 – 81.3) |
| Bright or possibly bright on T2 | 75.0 (56.6 – 88.6) | 28.0 (18.2 – 39.6) | 30.8 (20.8 – 42.2) | 72.4 (52.8 – 87.3) |
| Bright on PD | 96.9 (83.8 – 99.9) | 9.3 (3.8 – 18.3) | 31.3 (22.4 – 41.4) | 87.5 (47.4 – 99.7) |
| Bright or possibly bright on PD | 100 (89.1 – 100) | 6.7 (2.2 – 14.9) | 31.4 (22.6 – 41.1) | 100 (47.8 – 100) |
| **Lentiform nucleus signal** |  |  |  |  |
| Bright on T2 | 62.5 (43.7 – 78.9) | 42.7 (31.3 – 54.6) | 31.8 (20.6 – 44.7) | 72.7 (57.2 – 85.0) |
| Bright or possibly bright on T2 | 90.6 (75.0 – 98.0) | 22.7 (13.8 – 33.8) | 33.3 (23.6 – 44.3) | 85.0 (62.1 – 96.8) |
| Bright on PD | 84.4 (67.2 – 94.7) | 8.0 (3.0 – 16.6) | 28.1 (19.4 – 38.2) | 54.6 (23.4 – 83.3) |
| Bright or possibly bright on PD | 96.9 (83.8 – 99.9) | 5.3 (1.5 – 13.1) | 30.4 (21.7 – 40.3) | 80.0 (28.4 – 99.5) |
| **Pulvinar signal** |  |  |  |  |
| Bright on T2 | 46.9 (29.1 – 65.3) | 74.7 (63.3 – 84.0) | 44.1 (27.2 – 62.1) | 76.7 (65.4 – 85.8) |
| Bright or possibly bright on T2 | 62.5 (43.7 – 78.9) | 62.7 (50.7 – 73.6) | 41.7 (27.6 – 56.8) | 79.7 (67.2 – 89.0) |
| Bright on PD | 93.8 (79.2 – 99.2) | 28.0 (18.2 – 39.6) | 35.7 (25.6 – 46.9) | 91.3 (72.0 – 98.9) |
| Bright or possibly bright on PD | 96.9 (83.8 – 99.9) | 18.7 (10.6 – 29.3) | 33.7 (24.2 – 44.3) | 93.3 (68.1 – 99.8) |
| **Pulvinar sign** |  |  |  |  |
| Present on T2 | 18.7 (7.2 – 36.4) | 97.3 (90.7 – 99.7) | 75.0 (34.9 – 96.8) | 73.4 (63.9 – 82.1) |
| Present or same signal as putamen on T2 | 62.5 (43.7 – 78.9) | 66.7 (54.8 – 77.1) | 44.4 (29.6 – 60.0) | 80.7 (68.6 – 89.6) |
| Present on PD | 65.6 (46.8 – 81.4) | 29.3 (19.4 – 41.0 | 28.4 (18.5 – 40.1) | 66.7 (48.2 – 82.0) |
| Present or same signal as putamen on PD | 90.6 (75.0 – 98.0) | 26.7 (17.1 – 38.1) | 34.5 (24.5 – 45.7) | 87.0 (66.4 – 97.2) |

vCJD = variant Creutzfeldt-Jakob disease, CI = confidence interval, PD = proton density, - values cannot be calculated

| **Predicting sCJD** | Sensitivity %(95% CI) | Specificity %(95% CI) | Positive predictive value(95% CI) | Negative predictive value(95% CI) |
| --- | --- | --- | --- | --- |
| Brain atrophy | 14.7 (7.6 – 24.7) | 100 (89.1 – 100) | - | - |
| White matter hyperintensities | 20.0 (11.7 – 30.8) | 100 (89.1 – 100) | - | - |
| **Caudate nucleus signal** |  |  |  |  |
| Bright on T2 | 52.0 (40.2 – 63.7) | 50.0 (31.9 – 68.1) | 70.9 (57.1 – 82.4) | 30.8 (18.7 – 45.1) |
| Bright or possibly bright on T2 | 72.0 (60.4 – 81.8) | 25.0 (11.5 – 43.4) | 69.2 (57.8 – 79.2) | 27.6 (12.7 – 47.2) |
| Bright on PD | 90.7 (81.7 – 96.2) | 3.1 (0.1 – 16.2) | 68.7 (58.6 – 77.6) | 12.5 (0.3 – 52.7) |
| Bright or possibly bright on PD | 93.3 (85.1 – 97.8) | 0.0 (0.0 – 10.9) | 68.6 (58.7 – 77.5) | 0.0 (0.0 – 52.2) |
| **Lentiform nucleus signal** |  |  |  |  |
| Bright on T2 | 57.3 (45.4 – 68.7) | 37.5 (21.1 – 56.3) | 68.3 (55.3 – 79.4) | 27.3 (15.0 – 42.8) |
| Bright or possibly bright on T2 | 77.3 (66.2 – 86.2) | 9.4 (2.0 – 25.0) | 66.7 (55.8 – 76.4) | 15.0 (3.2 – 37.9) |
| Bright on PD | 92.0 (83.4 – 97.0) | 15.6 (5.3 – 32.8) | 71.9 (61.8 – 80.6) | 45.5 (16.8 – 76.6) |
| Bright or possibly bright on PD | 94.7 (86.9 – 98.5) | 3.1 (0.1 – 16.2) | 69.6 (59.7 – 78.3) | 20.0 (0.5 – 71.6) |
| **Pulvinar signal** |  |  |  |  |
| Bright on T2 | 25.3 (16.0 – 36.7) | 53.1 (34.7 – 70.9) | 55.9 (37.9 – 72.8) | 23.3 (14.2 – 34.7) |
| Bright or possibly bright on T2 | 37.3 (26.4 – 49.3) | 37.5 (21.1 – 56.3) | 58.3 (43.2 – 72.4) | 20.3 (11.0 – 32.8) |
| Bright on PD | 72.0 (60.4 – 81.8) | 6.3 (0.8 – 20.8) | 64.3 (53.1 – 74.5) | 8.7 (1.1 – 28.0) |
| Bright or possibly bright on PD | 81.3 (70.7 – 89.4) | 3.1 (0.1 – 16.2) | 66.3 (55.7 – 75.8) | 6.7 (0.2 – 32.0) |
| **Pulvinar sign** |  |  |  |  |
| Present on T2 | 2.7 (0.3 – 9.3) | 81.3 (63.6 – 92.8) | 25.0 (3.2 – 65.1) | 26.3 (17.9 – 36.1) |
| Present or same signal as putamen on T2 | 33.3 (22.9 – 45.2) | 37.5 (21.1 – 56.3) | 55.6 (40.0 – 70.4) | 19.4 (10.4 – 31.4) |
| Present on PD | 70.7 (59.0 – 80.6) | 34.4 (18.6 – 53.2) | 71.6 (60.0 - 81.5) | 33.3 (18.0 – 51.8) |
| Present or same signal as putamen on PD | 73.3 (61.9 – 82.9) | 9.4 (2.0 – 25.0) | 65.5 (54.3 – 75.5) | 13.0 (2.8 – 33.6) |

sCJD = sporadic Creutzfeldt-Jakob disease, CI = confidence interval, PD = proton density, - values cannot be calculated
