# Supplementary material for: Post-mortem magnetic resonance imaging in patients with suspected prion disease: Pathological confirmation, sensitivity, specificity and observer reliability. A national registry
Source: PLoS One. 2018 Aug 7;13(8):e0201434. doi: 10.1371/journal.pone.0201434 (PMC6080765; doi:10.1371/journal.pone.0201434)
Supplement: S3 Table — PD = proton density, CJD = Creutzfeldt-Jakob Disease a The second reader did not provide a rating for pulvinar signal on T2 or PD for one case each; denominator is 199. (DOCX) [file pone.0201434.s003.docx]

**S3 Table: Interobserver agreement**

|  | **Total cases agreed**  **n (%)** | **Agreed present**  **n (%)** | **Agreed absent**  **n (%)** | **Disagreed**  **n (%)** |
| --- | --- | --- | --- | --- |
| Brain atrophy | 169 (84.5) | 32 (16.0) | 137 (68.5) | 31 (15.5) |
| White matter hyperintensities | 168 (84.0) | 31 (15.5) | 137 (68.5) | 32 (16.0) |
| **Caudate nucleus signal** |  |  |  |  |
| Bright on T2 | 139 (69.5) | 18 (9.0) | 121 (60.5) | 61 (30.5) |
| Bright or possibly bright on T2 | 117 (58.0) | 29 (14.5) | 88 (44.0) | 83 (41.5) |
| Bright on PD | 50 (25.0) | 37 (18.5) | 13 (6.5) | 150 (75.0) |
| Bright or possibly bright on PD | 56 (28.0) | 49 (24.5) | 7 (3.5) | 144 (72.0) |
| **Lentiform nucleus signal** |  |  |  |  |
| Bright on T2 | 146 (73.0) | 36 (18.0) | 110 (55.0) | 54 (27.0) |
| Bright or possibly bright on T2 | 136 (68.0) | 66 (33.0) | 70 (35.0) | 64 (32.0) |
| Bright on PD | 76 (38.0) | 52 (26.0) | 24 (12.0) | 124 (62.0) |
| Bright or possibly bright on PD | 88 (44.0) | 78 (39.0) | 10 (5.0) | 112 (56.0) |
| **Pulvinar signal^a^** |  |  |  |  |
| Bright on T2 | 152 (76.4) | 7 (3.5) | 145 (72.9) | 47 (23.6) |
| Bright or possibly bright on T2 | 140 (70.4) | 15 (7.5) | 125 (62.8) | 59 (29.7) |
| Bright on PD | 72 (36.2) | 14 (7.0) | 58 (29.2) | 127 (63.8) |
| Bright or possibly bright on PD | 66 (33.2) | 27 (13.6) | 39 (19.6) | 133 (66.8) |
| **Pulvinar sign** |  |  |  |  |
| Present on T2 | 168 (84.0) | 3 (1.5) | 165 (82.50) | 32 (16.0) |
| Present or same signal as putamen on T2 | 127 (63.5) | 14 (7.0) | 113 (56.5) | 73 (36.5) |
| Present on PD | 174 (87.0) | 2 (1.0) | 172 (86.0) | 26 (13.0) |
| Present or same signal as putamen on PD | 75 (37.5) | 21 (10.5) | 54 (27.0) | 125 (62.5) |
| **Final diagnosis** |  |  |  |  |
| CJD | 102 (51.0) | 53 (26.5) | 49 (24.5) | 98 (49.0) |

PD = proton density, CJD = Creutzfeldt-Jakob Disease

**^a^** The second reader did not provide a rating for pulvinar signal on T2 or PD for one case each; denominator is 199.
