# Supplementary material for: Post-mortem magnetic resonance imaging in patients with suspected prion disease: Pathological confirmation, sensitivity, specificity and observer reliability. A national registry
Source: PLoS One. 2018 Aug 7;13(8):e0201434. doi: 10.1371/journal.pone.0201434 (PMC6080765; doi:10.1371/journal.pone.0201434)
Supplement: S1 File — (DOCX) [file pone.0201434.s004.docx]

**S1 File: Imaging coding for imaging readers**

| Question | | Choice of responses | | | |
| --- | --- | --- | --- | --- | --- |
| Is there generalised atrophy? | | Yes | | No | |
| Are there white matter signal hyperintensities? | | Yes | | No | |
| Caudate nucleus signal compared to expected | T2 | Normal | Possibly bright | | Bright |
|  | PD | Normal | Possibly bright | | Bright |
| Lentiform nucleus signal compared to expected | T2 | Normal | Possibly bright | | Bright |
|  | PD | Normal | Possibly bright | | Bright |
| Pulvinar signal compared to expected | T2 | Normal | Possibly bright | | Bright |
|  | PD | Normal | Possibly bright | | Bright |
| Is the pulvinar bright compared to other basal ganglia? | T2 | Yes | No | | Same |
|  | PD | Yes | No | | Same |
| Do you think this patient has CJD? |  | Not CJD | Yes, variant CJD | | Yes, sporadic CJD |
